# *FCER1A* Downregulation in Infectious Pneumonia: A Multi-Modal Study Combining Bioinformatics, Animal Models, and Reverse Pharmacology

**DOI:** 10.3390/genes16111294

**Published:** 2025-10-31

**Authors:** Yuan Cai, Xiaolong Feng, Mengxiong Xiao, Qian Li, Xinru Tao, Penghui Li

**Affiliations:** 1 Institute of Innovative Chinese Medicine, Hunan Academy of Chinese Medicine, Changsha 410013, China; tcmyuanyuan@163.com (Y.C.);; 2 Experimental Research Center, China Academy of Chinese Medical Sciences, Beijing 100700, China; 3 College of Traditional Chinese Medicine and Health, Nanfang College Guangzhou, Guangzhou 510970, China

**Keywords:** infectious pneumonia, bioinformatics, machine learning, reverse network pharmacology, *FCER1A*

## Abstract

**Background**: Infectious pneumonia remains a major global health challenge with high morbidity and mortality, especially among vulnerable groups. Current diagnostic approaches lack sufficient specificity and accuracy. This study aimed to identify core diagnostic genes, explore their biological functions, and predict potential natural compounds targeting these genes to improve diagnostic and therapeutic strategies. **Methods**: Gene expression profiles from the GEO database (GSE103119) were analyzed to identify differentially expressed genes (DEGs). Hub genes were selected by integrating protein–protein interaction (PPI) networks and multiple machine learning algorithms. Expression patterns of the identified hub gene were validated in a murine pneumonia model. Reverse network pharmacology was applied to screen natural compounds, followed by molecular docking and molecular dynamics simulations to evaluate binding affinity and complex stability. **Results**: A total of 2550 DEGs were identified. *FCER1A* was consistently determined as a hub gene through PPI and machine learning analyses, showing significant downregulation in infectious pneumonia patients. Animal experiments confirmed pronounced reduction of *Fcer1a* transcription in both lung tissue and whole blood of pneumonia model mice. Two natural compounds, pyrogallol and tectorigenin, were identified as potential ligands for *FCER1A*. Molecular simulations confirmed stable binding with the target protein, with tectorigenin exhibiting superior binding affinity. **Conclusions**: This study proposes *FCER1A* as a promising diagnostic biomarker for infectious pneumonia and suggests tectorigenin as a candidate compound for further therapeutic development.

## 1. Introduction

Infectious pneumonia remains a leading global cause of respiratory infection-related morbidity and mortality, imposing a persistent and substantial burden on public health systems [1]. Among these, community-acquired pneumonia (CAP) is a critical contributor to infection-related mortality, exerting particularly pronounced clinical impacts on vulnerable populations such as children, the elderly, and immunocompromised individuals [2,3]. Distinct age-based disparities in pathogen susceptibility have been demonstrated: an epidemiological study on severe community-acquired pneumonia (SCAP) revealed that respiratory syncytial virus (RSV, 21.30%) and Streptococcus pneumoniae (12.61%) were the most prevalent pathogens in pediatric populations, whereas influenza virus (10.94%) and Pseudomonas aeruginosa (*P. aeruginosa*, 15.37%) predominated in elderly cohorts [4]. These findings underscore the population-specific distribution of pneumonia pathogens and suggest that age and underlying health status may play pivotal roles in pathogen selection and infection severity, thereby providing critical epidemiological evidence for formulating targeted prevention strategies across diverse populations.

The current clinical diagnosis of infectious pneumonia primarily relies on clinical evaluation, imaging studies, laboratory tests, and microbiological assays [5,6]. However, existing diagnostic methods exhibit significant limitations in specificity, non-invasiveness, and accuracy, often leading to a reliance on empirical therapy in clinical practice. Consequently, the identification of novel and effective biomarkers for infectious pneumonia is crucial for its timely diagnosis and management.

The pathogenesis of infectious pneumonia is closely associated with immune dysregulation [7]. Upon pathogen invasion, the host initiates a complex response involving both innate and adaptive immunity [8]. Neutrophils and macrophages are rapidly recruited to the lungs to phagocytose pathogens, while T lymphocytes (including CD4^+^ and CD8^+^ T cells) and natural killer (NK) cells mediate the specific clearance of pathogens and regulate inflammatory homeostasis [7]. However, excessive or dysregulated immune activation—characterized by aberrant infiltration of pro-inflammatory cells such as M0 macrophages and neutrophils—often leads to tissue damage, cytokine storm, and progressive pulmonary dysfunction [9]. Although previous studies have elucidated the critical role of immune cell infiltration in pneumonia, the key molecular targets regulating this immune imbalance remain elusive.

Advances in high-throughput sequencing and bioinformatics have established public databases, such as the Gene Expression Omnibus (GEO), as invaluable resources for identifying disease-associated molecular signatures [10]. Integrated analysis of GEO datasets enables the systematic identification of core genes with diagnostic or prognostic value [11]. Machine learning algorithms—including Least Absolute Shrinkage and Selection Operator (LASSO) regression, Support Vector Machine-Recursive Feature Elimination (SVM-RFE), and random forests—offer robust tools for screening potential biomarkers by reducing dimensionality and enhancing model generalizability, thereby overcoming the limitations of conventional single-gene analyses [12,13]. However, most current bioinformatics studies on infectious pneumonia lack experimental validation in animal models, which constrains the translational potential of the identified biomarkers.

**Figure 1:**
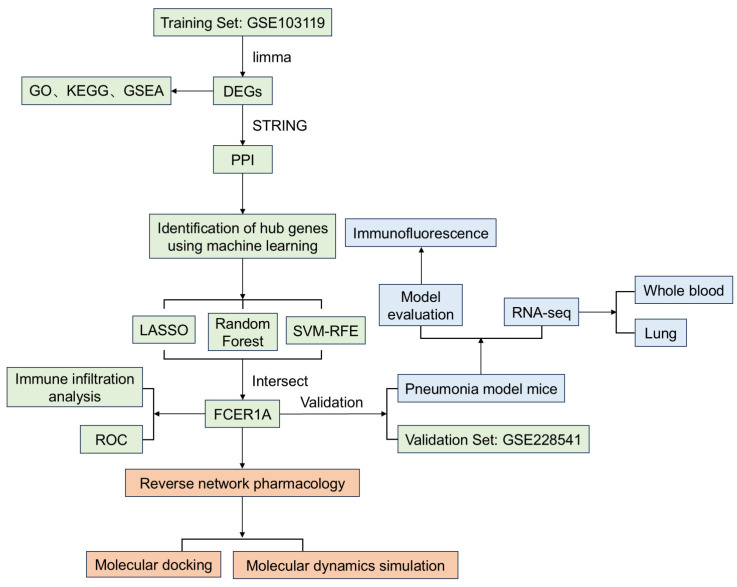
The workflow of the research. This study firstly identified and validated core genes for infectious pneumonia by integrating bioinformatics with machine learning (green). Subsequently, the expression of core gene was further confirmed through animal experiments (blue). Finally, potential bioactive compounds were predicted by employing a reverse network pharmacology approach (orange).

To address these research gaps, this study integrated multi-dimensional methodologies encompassing bioinformatics, animal experimentation, and computational biology. First, utilizing two independent GEO datasets (training set GSE103119 and validation set GSE228541), we identified differentially expressed genes (DEGs) between infectious pneumonia patients and healthy controls, constructed a protein–protein interaction (PPI) network, and employed three machine learning algorithms to screen for core diagnostic genes. Subsequently, we established a Pseudomonas aeruginosa-induced pneumonia model in BALB/c mice to validate the expression of the core gene, Fc epsilon receptor Ia (*FCER1A*), and to analyze its associated pathological, inflammatory, and immunological alterations. Finally, we employed reverse network pharmacology to predict natural compounds targeting *FCER1A*, and utilized molecular docking and molecular dynamics simulations to evaluate their binding modes and stability with *FCER1A*. This study aims to provide novel biomarkers and potential adjuvant therapeutic candidates for the diagnosis and treatment of infectious pneumonia, thereby laying a foundation for subsequent translational research and clinical applications. The research workflow is illustrated in Figure 1.

## 2. Materials and Methods

### 2.1. Processing of Data

The data were sourced from the Gene Expression Omnibus (GEO, http://www.ncbi.nlm.nih.gov/geo (accessed on 04 August 2025)), a public functional genomics repository maintained by the National Center for Biotechnology Information (NCBI). This international public database archives a vast array of gene expression profiles generated through high-throughput sequencing and microarray technologies. Through systematic retrieval using keywords such as “infectious pneumonia” and “pneumonia”, two datasets meeting the study criteria were selected: GSE103119 and GSE228541. The GSE103119 dataset comprised whole-blood mRNA expression profiles from 152 infectious pneumonia patients and 20 healthy controls, while GSE228541 contained whole-blood mRNA expression data from 14 infectious pneumonia patients and 15 healthy controls. GSE103119 was designated as the internal training set, and GSE228541 served as the external validation set for subsequent analyses. Raw CEL files were processed using the “affy” R package, with background correction, quantile normalization, and probe-level data summarization performed via the Robust Multi-array Average (RMA) algorithm. Probe identifiers were mapped to official gene symbols based on platform-specific annotation files. Probes that failed to match any gene symbol were excluded from further analysis. For multiple probes corresponding to the same gene, the average expression value across all probes was calculated as the final expression value for downstream analyses. A comprehensive description of the demographic and clinical features for the GSE103119 and GSE228541 cohorts can be found in the Supplementary Methods.

### 2.2. Identification of DEGs

Differential expression analysis was performed using the “limma” R package to identify DEGs between whole blood samples from infectious pneumonia patients and healthy controls. The filtering thresholds for DEG identification were set as |log_2_(fold change)| > 1 and an adjusted *p*-value < 0.05 after false discovery rate (FDR) correction. Genes meeting these criteria were considered statistically significant DEGs and were subsequently subjected to functional enrichment and pathway analyses [14].

### 2.3. Functional Enrichment Analysis of DEGs

Functional enrichment analysis of DEGs was conducted using the clusterProfiler R package with the org.Hs.eg.db human gene annotation database, employing a statistical significance threshold of adjusted *p*-value < 0.05.

### 2.4. Construction of the PPI Network

To identify core targets among the DEGs, this study conducted a PPI network analysis of the encoded proteins using the STRING database (https://string-db.org/ (accessed on 10 August 2025)). The analysis was restricted to “Homo sapiens” with a minimum interaction confidence score threshold > 0.9 to ensure high-reliability interactions. The resulting PPI network data were exported in TSV format and subsequently imported into Cytoscape software (v3.10.1) for visualization and topological analysis. Isolated nodes without connections were removed from the network, while targets demonstrating significant interactions were retained and identified as core targets for subsequent in-depth analysis.

### 2.5. Machine Learning

The rapid advancement of artificial intelligence technology has significantly enhanced its application in screening novel diagnostic biomarkers for diseases. This study employed three machine learning approaches—LASSO regression, SVM-RFE, and random forest—to identify hub genes associated with infectious pneumonia diagnosis. The GSE103119 dataset served as the training set, while the GSE228541 dataset was utilized as an external validation set. In the LASSO regression model, the response variable was assumed to follow a binomial distribution, with the optimal lambda value (lambda.min) determined through 10-fold cross-validation to select feature genes. The SVM-RFE algorithm employed 5-fold cross-validation to evaluate and rank feature importance, thereby identifying the optimal feature subset. The random forest model constructed multiple decision trees using ensemble learning, calculating the Mean Decrease Accuracy for each gene to assess importance, with key genes ultimately selected based on feature importance rankings. These integrated approaches enhanced the robustness and interpretability of feature selection, establishing a reliable computational framework for identifying diagnostic biomarkers of infectious pneumonia [15]. The detailed analysis workflow of machine learning is available in the Methods section of the Supplementary Materials.

### 2.6. Construction of the Receiver Operating Characteristic (ROC) Curve

To evaluate the accuracy of the constructed model and the diagnostic value of hub genes, this study utilized the pROC package to generate ROC curves and calculated metrics including area under the curve (AUC), specificity, and sensitivity for systematically assessing the discriminatory power of hub genes in infectious pneumonia. The reliability and generalizability of the selected biomarker genes were further validated using an independent external dataset (GSE228541) to verify their diagnostic capability.

### 2.7. Immune Cell Infiltration ANALYSIS

Immune infiltration analysis was performed using the CIBERSORT R function with deconvolution methodology to comprehensively evaluate differences in immune cell infiltration between pneumonia patients and healthy controls, with box plots visualizing the immune cell composition across both sample groups. Differences in immune cell proportions were assessed using the Wilcoxon rank-sum test. Results with *p* < 0.05 were considered statistically significant [16].

### 2.8. Establishment of a P. aeruginosa-Induced Infectious Pneumonia Model in BALB/c Mice

The standard strain of *P. aeruginosa* (CMCC (B) 10104) was purchased from the National Institutes for Food and Drug Control. (Beijing, China). Thirty 7–8-week-old SPF BALB/c mice were supplied by Hunan Silaike Jingda Experimental Animal Co., Ltd. (Changsha, China). After one week of acclimatization under standard laboratory conditions, all mice were randomly assigned to two groups: a normal control group (Control) and a pneumonia model group (Pneumonia). Mice in the Pneumonia group received slow intranasal instillation of 50 μL of a *P. aeruginosa* bacterial suspension (5 × 10^9^ CFU/mL), while the Control group received an equal volume of PBS solution administered identically. Following infection, mice were continuously monitored for 30 h to record survival status and calculate survival rates. After surviving for 30 h, mice were deeply anesthetized by an intraperitoneal injection of sodium pentobarbital (150 mg/kg) and subsequently euthanized via cervical dislocation. Blood and lung tissue samples were collected immediately thereafter. A portion of whole blood samples was treated with RNA stabilizer and stored at −80 °C; another portion was centrifuged at 4000 rpm to collect supernatant, which was then stored at −80 °C. Lung tissues were perfused with PBS, with one portion fixed in 4% paraformaldehyde and the remaining tissue rapidly frozen in liquid nitrogen before transfer to a −80 °C ultra-low temperature freezer for preservation. All experimental protocols were approved by the Institutional Animal Care and Use Committee of Hunan Ansenmei Pharmaceutical Research Co., Ltd. (Changsha, China). (Ethics Approval No. IACUC-KY2025001).

### 2.9. Histological Analyses

Formalin-fixed lung tissue specimens were dehydrated, embedded in paraffin, and sectioned. Tissue sections were baked at 60 °C for 1 h, deparaffinized in xylene, and subsequently rehydrated through a graded ethanol series. Following hematoxylin and eosin (H&E) staining, the sections were dehydrated, cleared in xylene, and mounted with neutral resin. Histopathological evaluation and image acquisition were finally performed under a light microscope.

### 2.10. Immunofluorescence Staining Analysis

A standard immunohistochemical procedure was performed on paraffin-embedded tissue sections. Following deparaffinization in xylene and rehydration through a graded ethanol series, antigen retrieval was conducted using pH 9.0 EDTA buffer. The sections were then treated with 3% H_2_O_2_ to block endogenous peroxidase activity and incubated with 10% goat serum to reduce nonspecific binding. Primary antibody incubation proceeded at 4 °C overnight, followed by application of HRP-conjugated secondary antibodies. Signal amplification was achieved using tyramide reagents (TYR-570/520), and nuclei were counterstained with DAPI. Finally, the sections were mounted with anti-fade medium and visualized by fluorescence/confocal microscopy.

### 2.11. Transcriptomics Analysis with RNA-Seq

Total RNA was extracted from whole blood and lung tissue samples using TRIzol reagent, with RNA concentration quantified using a NanoDrop ND-1000 spectrophotometer. The detailed procedures for subsequent sample data analysis are described in the Supplementary Methods section [17]. Differential expression analysis employed a significance threshold of *p* < 0.05 and |log_2_(Fold Change)| > 1.

### 2.12. Reverse Screening of Bioactive Compounds for Hub Targets

Hub targets were imported into the BATMAN-TCM database for matching, and the resulting targets and compounds were visualized using Cytoscape software version 3.10.1 [18].

### 2.13. Molecular Docking

The 3D structures of small molecule ligands were directly obtained from the PubChem database (http://pubchem.ncbi.nlm.nih.gov/ (accessed on 29 August 2025)) and saved in the MOL2 file format. A high-resolution crystal structure of the protein target was selected from the PDB (http://www.rcsb.org/ (accessed on 29 August 2025)) to serve as the docking receptor and was subsequently prepared using PyMOL software, which included the removal of water molecules and phosphate groups. Molecular docking simulations were carried out using AutoDock Vina 1.5.6. The resulting protein–ligand complexes were visualized using Discovery Studio 2019 to analyze the binding interactions [19,20].

### 2.14. Molecular Dynamics Simulation

This study employed the GROMACS 2022 software package to perform molecular dynamics simulations, with a total production time of 100 nanoseconds (ns). The detailed analytical methodology is described in the Supplementary Materials.

### 2.15. Statistical Analysis

All statistical analyses and data visualizations were performed using Origin 2021 software. Data are presented as mean ± standard deviation (Mean ± SD). Intergroup comparisons were conducted using one-way analysis of variance (ANOVA), followed by Tukey’s test for multiple comparisons. A threshold of *p* < 0.05 was considered statistically significant.

## 3. Results

### 3.1. Based on the Bioinformatics Analysis Results

#### 3.1.1. Screening of DEGs Associated with Infectious Pneumonia

**Figure 2:**
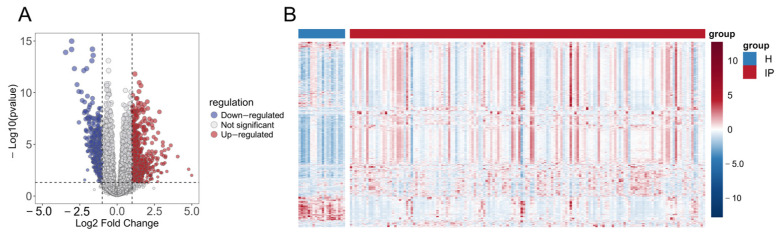
Identification of DEGs in infectious pneumonia. (**A**) Volcano plot shows the DEGs between the infectious pneumonia group and the healthy control group. (**B**) Heatmap shows the expression profiles of 2550 DEGs in the infectious pneumonia group and the healthy control group. The color gradient represents the normalized expression values (log_2_ fold change) of the differentially expressed genes, hereinafter the same. Note: H refers to the healthy control group; IP refers to the infectious pneumonia group; hereinafter the same.

Analysis of the GSE103119 dataset identified 2550 DEGs, comprising 1914 significantly upregulated and 636 significantly downregulated genes, with results visualized in a volcano plot (Figure 2A). A heatmap was subsequently generated to depict the expression patterns of these differentially expressed genes in infectious pneumonia patients versus healthy controls, demonstrating distinct expression profiles between the two groups (Figure 2B).

#### 3.1.2. Functional Enrichment Analysis Reveals Activated Immune and Inflammatory Pathways

**Figure 3:**
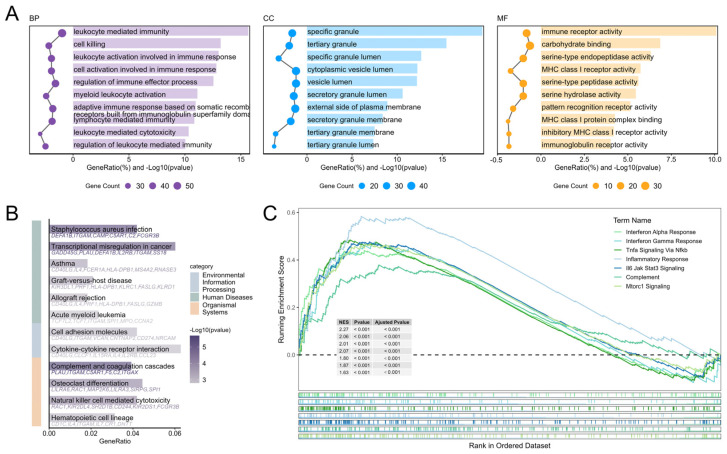
Functional enrichment analysis of DEGs. (**A**) GO functional enrichment analysis of DEGs. (**B**) KEGG functional enrichment analysis of DEGs. (**C**) Hallmark GSEA analysis of Whole-Genome Expression Profiles.

To gain deeper insights into the biological functions of differentially expressed genes, we conducted systematic functional enrichment analyses. GO analysis revealed significant enrichment of differentially expressed genes in immune-related functions: biological processes (BP) included leukocyte mediated immunity, cell killing, and lymphocyte mediated immunity; cellular components (CC) were primarily enriched in specific granules and tertiary granule lumens, suggesting activation of degranulation processes; while molecular functions (MF) were predominantly associated with immune receptor activity and serine-type endopeptidase activity (Figure 3A). KEGG pathway analysis further indicated significant enrichment in Staphylococcus aureus infection, complement and coagulation cascades, and cytokine-cytokine receptor interaction pathways, with notable activation of natural killer cell mediated cytotoxicity and cell adhesion molecules, demonstrating coordinated involvement of innate and adaptive immunity in combating pulmonary infection (Figure 3B). GSEA results additionally showed significant activation of gene sets associated with viral infection and inflammatory responses, including Interferon Alpha/Gamma Response, Tnfa Signaling via NFκB, Inflammatory Response, and Il6-Jak-Stat3 Signaling, further corroborating robust activation of immune and inflammatory signaling pathways during infection (Figure 3C).

#### 3.1.3. PPI Network Reveals Immune Dysregulation

**Figure 4:**
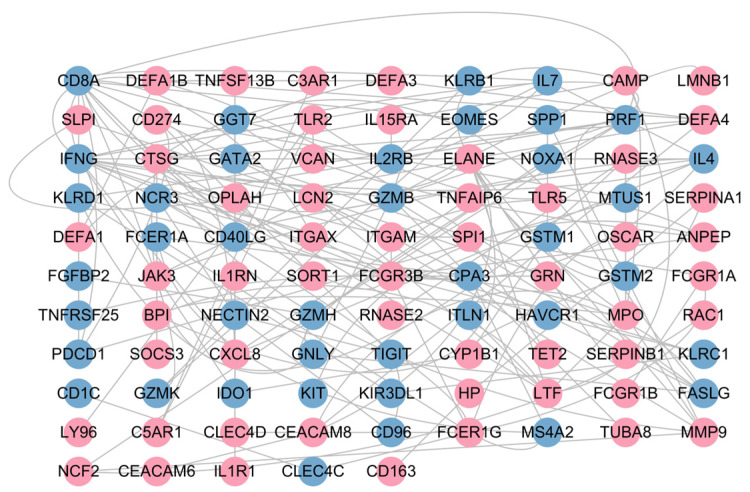
PPI network of DGEs. Nodes in red denote those significantly upregulated in the infectious pneumonia group, while those in blue indicate significant downregulation in this group.

To identify the core targets among differentially expressed genes, we constructed a PPI network based on the STRING database. After removing isolated nodes, a core PPI network diagram containing 95 nodes and 158 interaction edges was obtained (Figure 4). Analysis showed that there is significant immune response differentiation in infectious pneumonia. The expression of myeloid cell-mediated innate immunity-related genes was generally upregulated (e.g., DEFA1, DEFA3, CAMP, ITGAM, and CXCL8). In contrast, the expression of adaptive immune effector function-related genes was significantly downregulated (e.g., CD8A, PRF1, GZMB, FCER1A, and IFNG). This network reveals the molecular interaction basis of immune activation and regulatory imbalance in infectious pneumonia, providing a basis for further exploration of core targets.

#### 3.1.4. Identification of Hub Gene Using Machine Learning

**Figure 5:**
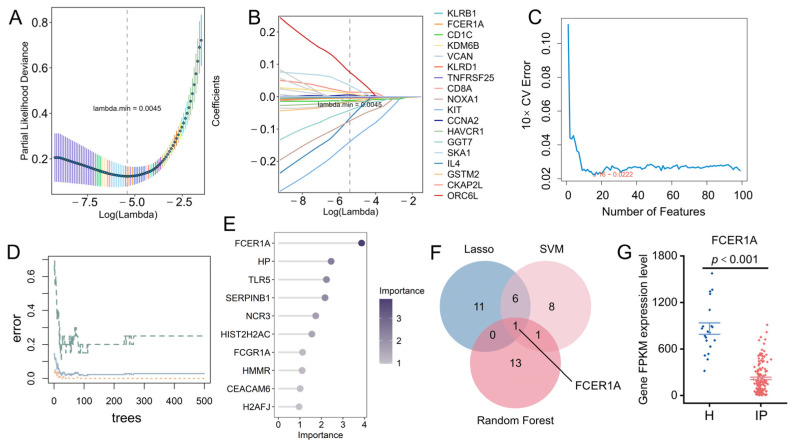
Identification of hub genes using machine learning. (**A**,**B**) 18 genes were identified through the LASSO regression algorithm. (**C**) Based on the feature selection error of the SVM-RFE algorithm, 13 genes were pinpointed. (**D**) Error curves of the random forest model as a function of the number of trees. (**E**) Feature importance of candidate genes derived from the random forest algorithm. (**F**) Venn diagram displaying the overlapping genes identified by the three machine learning models. (**G**) Expression profile of *FCER1A* in the GSE103119 dataset, presented as FPKM values.

To further identify diagnostic biomarkers capable of distinguishing infectious pneumonia patients from healthy controls, we employed three machine learning algorithms—LASSO regression, Random Forest, and SVM-RFE—to screen feature genes from the previously identified 95 core differentially expressed genes. The results demonstrated that LASSO regression identified 18 candidate genes (Figure 5A,B), SVM-RFE selected 16 genes (Figure 5C), and the Random Forest model screened 15 genes (Figure 5D,E). Intersection analysis of the results from the three algorithms revealed one overlapping gene: Fc Epsilon Receptor Ia (*FCER1A*) (Figure 5F). This gene exhibited significant downregulation in pneumonia patients (Figure 5G), suggesting its potential value as a diagnostic biomarker for infectious pneumonia.

#### 3.1.5. Expression of Hub Genes in the Validation Set and ROC Curve Analysis

**Figure 6:**
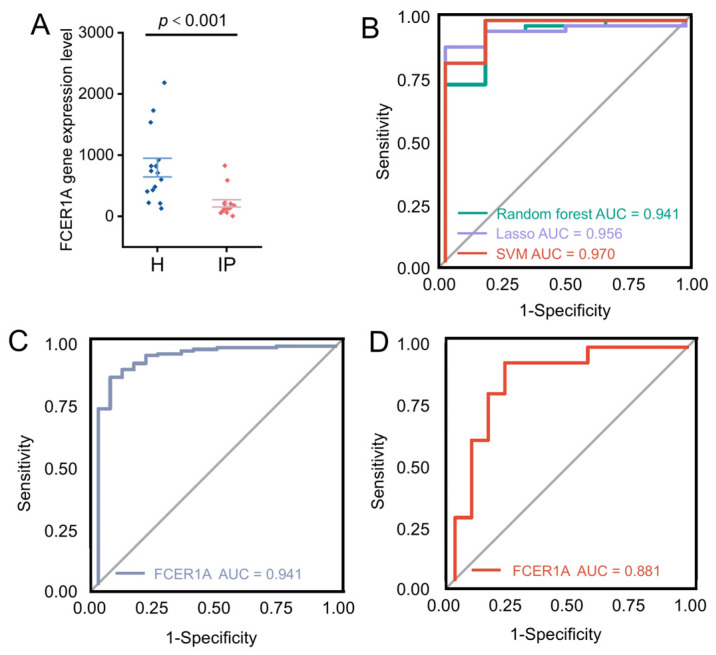
Validation of hub genes and evaluation via ROC curves. (**A**) Scatter plot illustrating the expression levels of the *FCER1A* gene in healthy control and infectious pneumonia groups, with a significant difference denoted by *p* < 0.001. (**B**) ROC curves for predictive models generated via the random forest (AUC = 0.941), LASSO (AUC = 0.956), and SVM (AUC = 0.970) algorithms. (**C**) ROC curve of the *FCER1A* gene as a predictor in the training set. (**D**) ROC curve of the *FCER1A* gene as a predictor in the validation set.

To further evaluate the discriminatory performance of the three machine learning models in an external validation set, ROC curves were constructed and the AUC was calculated. Results demonstrated that hub gene expression levels were significantly lower in infectious pneumonia patients compared to healthy controls in the GSE228541 validation set (*p* < 0.001; Figure 6A). The three machine learning models achieved AUC values ranging from 0.94 to 0.97 in the validation set, indicating excellent generalization performance (Figure 6B). Notably, *FCER1A* achieved AUC values of 0.941 in the training set (GSE103119; Figure 6C) and 0.881 in the validation set (GSE228541; Figure 6D), further demonstrating its robust predictive capability in distinguishing infectious pneumonia patients from healthy individuals.

#### 3.1.6. Immune Cell Infiltration Analysis

**Figure 7:**
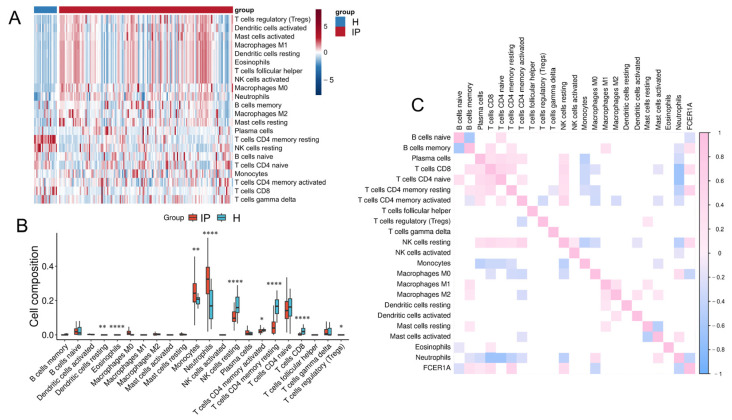
Immune cell infiltration analysis. (**A**) Heatmap depicting the infiltration levels of various immune cell subsets in the healthy control group and the infectious pneumonia group. (**B**) Boxplots illustrating the distribution of immune cell composition across the healthy control group and the infectious pneumonia group, as predicted by CIBERSORT analysis. (**C**) Correlation heatmap showing associations between *FCER1A* gene expression and infiltration levels of distinct immune cell subsets. * *p* < 0.05, ** *p* < 0.01 **** *p* < 0.0001 vs. healthy control group.

Infectious pneumonia can induce both pulmonary and systemic inflammatory responses. To investigate differences in whole-blood immune cell composition between infectious pneumonia patients and healthy controls, we performed a comparative analysis of their immune microenvironments using the CIBERSORT algorithm (which provides computational estimates of immune cell fractions from gene expression data) (Figure 7A). Evaluation of 22 immune cell subset proportions revealed significant differences in eight immune cell types between the two groups (Figure 7B). Specifically, pneumonia patients exhibited elevated proportions of Macrophages M0, Monocytes, Neutrophils, and regulatory T cells (Tregs) in peripheral blood, alongside reduced proportions of resting NK cells, activated CD4 memory T cells, resting CD4 memory T cells, and CD8 T cells. Further analysis revealed significant correlations among different immune cell types in both pneumonia patients and healthy controls. Neutrophils showed negative correlations with CD4 naïve T cells, CD8 T cells, resting NK cells, and resting CD4 memory T cells. The hub gene *FCER1A* demonstrated negative correlations with Macrophages M0 and Neutrophils, but positive correlations with memory B cells, CD8 T cells, resting CD4 memory T cells, and resting NK cells (Figure 7C). These findings indicate that *FCER1A* is closely associated with multiple immune cell subsets, providing important insights for further investigation into the immunoregulatory mechanisms of infectious pneumonia.

### 3.2. Based on Animal Experiment Results

#### 3.2.1. Changes in Pathological Indicators of *P. aeruginosa*-Induced Acute Infectious Pneumonia Mouse Model

**Figure 8:**
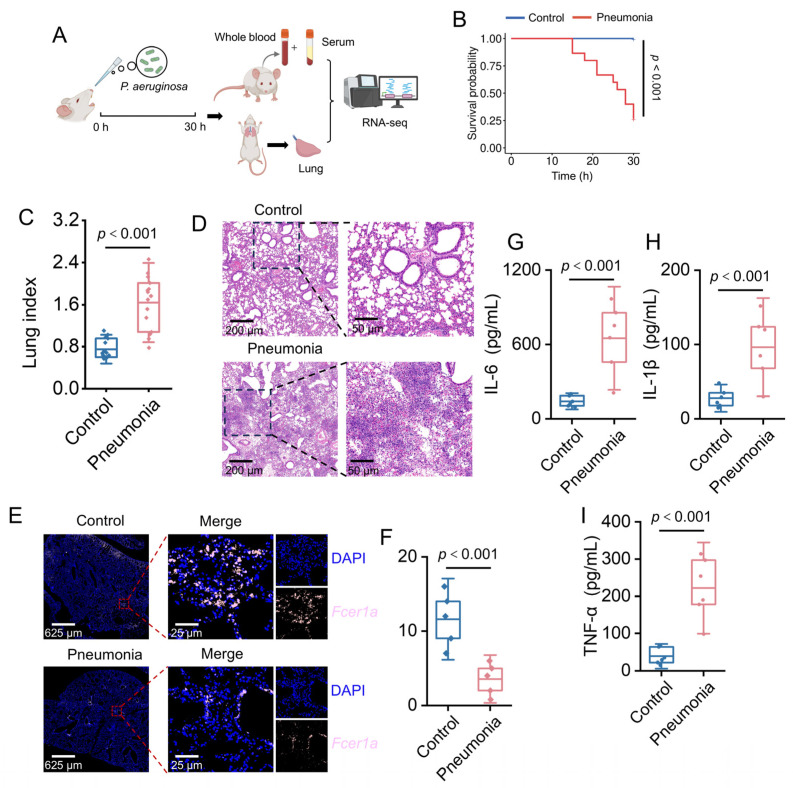
Characterization of Pseudomonas aeruginosa-induced pneumonia in a murine model. (**A**) Schematic of the experimental design. (**B**) Kaplan–Meier survival curves for mice in Control and Pneumonia groups (*p* < 0.001). (**C**) Violin plot depicting the lung index in Control and Pneumonia groups (*p* < 0.001). (**D**) HE-stained lung tissue sections from Control and Pneumonia groups. (**E**) Results of immunofluorescence staining for *Fcer1a* (pink). (**F**) Relative fluorescence intensity of *Fcer1a* in lung tissues (*p* < 0.001). (**G**–**I**) Box plots of serum proinflammatory cytokine concentrations (IL-6, IL-1β, TNF-α) in Control and Pneumonia groups (*p* < 0.001).

To validate the expression changes of *Fcer1a* in an animal model, an acute pneumonia mouse model was established via intranasal instillation of *P. aeruginosa* (Figure 8A). At 15 h post-infection, control group mice exhibited no significant abnormalities, whereas the pneumonia group displayed typical signs of infection, including piloerection, tachypnea, reduced activity, and conjunctival hyperemia. By 30 h post-infection, the survival rate of the pneumonia group declined to 26.67% (Figure 8B). Lung organ coefficient analysis revealed a significantly higher lung index in the pneumonia group compared to the control group (*p* < 0.001, Figure 8C), indicating successful establishment of the pneumonia model. Histopathological evaluation showed intact lung tissue structure with no significant inflammatory infiltration or tissue damage in the control group. In contrast, the pneumonia group exhibited marked pathological alterations, including extensive bronchial epithelial denudation, substantial neutrophil infiltration in alveolar spaces, alveolar septal rupture, and perivascular inflammatory cell infiltration (Figure 8D). Immunofluorescence staining demonstrated that *Fcer1a* protein expression and distribution in lung tissues were significantly higher in the normal group than in the pneumonia group, consistent with expectations (Figure 8E,F). Serum inflammatory cytokine assays indicated significantly elevated levels of TNF-α, IL-1β, and IL-6 in the pneumonia group compared to the control group (*p* < 0.001, Figure 8G–I), suggesting that pathogen infection triggered a systemic inflammatory response.

#### 3.2.2. Whole Blood and Lung Tissue Transcriptome Analysis of Acute Pneumonia Mice

**Figure 9:**
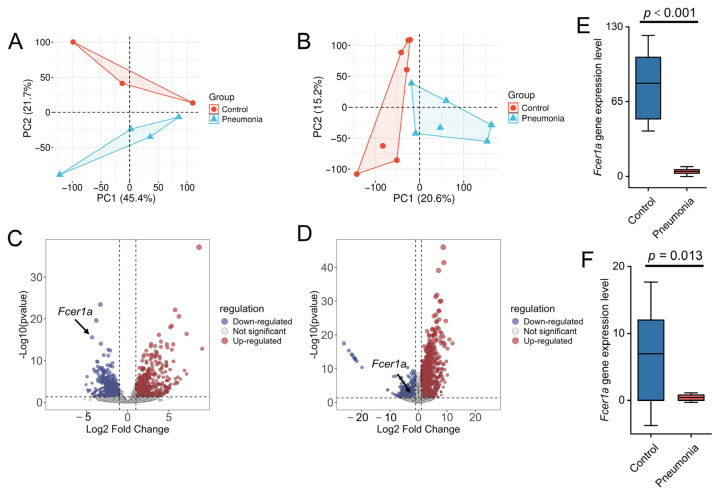
Transcriptomic profiling and *Fcer1a* expression analysis in control and pneumonia groups. PCA of the transcriptome from whole blood (**A**) and lung tissue (**B**). Volcano plots of DEGs from the whole blood transcriptome (**C**) and lung tissue transcriptome (**D**). Gene expression levels of *Fcer1a* in the whole blood transcriptome (**E**) and lung tissue transcriptome (**F**).

To investigate the transcriptional expression of *Fcer1a*, transcriptomic sequencing was performed on whole blood and lung tissue samples from mice. Principal component analysis (PCA) revealed a clear separation between the control and model groups at the transcriptional level (Figure 9A,B). Analysis using the limma package identified 997 DEGs in whole blood (548 upregulated and 449 downregulated) and 1982 DEGs in lung tissue (1360 upregulated and 622 downregulated). The results are presented as volcano plots, with the key gene *Fcer1a* highlighted (Figure 9C,D). Expression level analysis demonstrated that *Fcer1a* transcript levels were significantly higher in the normal group compared to the model group in both whole blood and lung tissue samples (*p* < 0.05; Figure 9E,F). Collectively, these results indicate that the transcriptional expression of the key gene *Fcer1a* is significantly downregulated in mice with infectious pneumonia, consistent with prior predictions, and suggest a crucial role for *Fcer1a* in the immunomodulatory mechanisms of infectious pneumonia.

### 3.3. Reverse Network Pharmacology Results Analysis

#### 3.3.1. Bioactive Component Prediction Results

**Figure 10:**
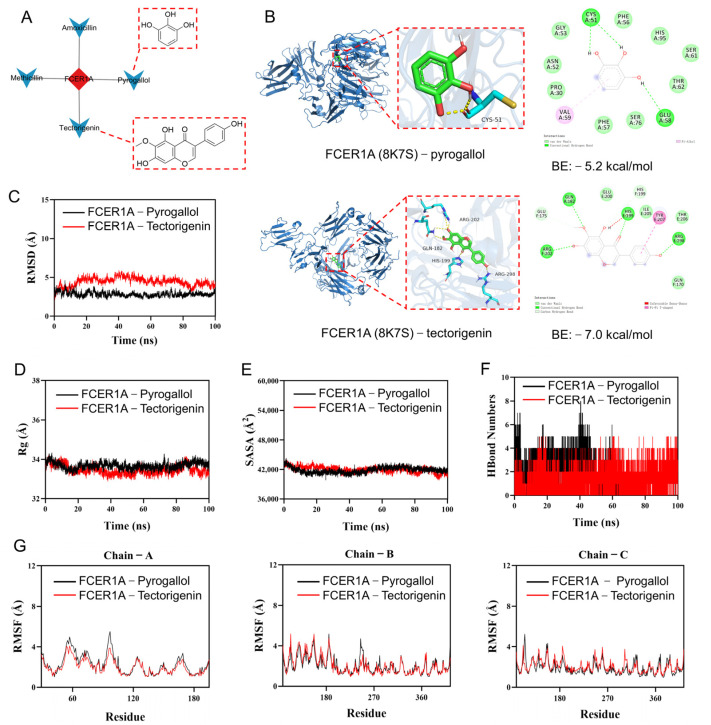
Screening of active compounds via reverse network pharmacology, coupled with molecular docking and molecular dynamics simulation. (**A**) Target-compound network diagram and molecular structures of active compounds. (**B**) Molecular docking results. (**C**) Root-mean-square deviation (RMSD) values of the protein–ligand complex over time. (**D**) Radius of gyration (Rg) values of the protein–ligand complex over time. (**E**) Solvent-accessible surface area (SASA) values of the protein–ligand complex over time. (**F**) Hydrogen bond (HBonds) counts of the protein–ligand complex over time. (**G**) Root-mean-square fluctuation (RMSF) values of each residue in the protein–ligand complex.

The target FCER1A was input into the BATMAN-TCM database for analysis. This database is an integrated platform combining systems biology and pharmacology, used to predict the molecular mechanisms of traditional Chinese medicine [21]. A “target-compound” interaction network was constructed (Figure 10A) to screen for candidate compounds potentially acting on this target. Four compounds were screened out: pyrogallol (enrichment ratio: 3169), methicillin (enrichment ratio: 2717), tectorigenin (enrichment ratio: 1463), and amoxicillin (enrichment ratio: 679). Literature review indicated that methicillin and amoxicillin have a long research history with well-defined targets, and extensive basic and clinical studies have confirmed their efficacy against various common bacterial infections; thus, they were excluded from subsequent investigation. Existing studies demonstrate that pyrogallol can prevent influenza-induced lung injury [22], disrupt staphylococcal biofilms, and treat inflammatory diseases [23]; tectorigenin possesses broad-spectrum antimicrobial, hepatoprotective, and anti-inflammatory activities, among others [24]. However, no direct evidence currently indicates a definitive ameliorative effect of either compound on infectious pneumonia. Based on their notable antimicrobial and anti-inflammatory activities, we hypothesize that pyrogallol and tectorigenin may exert adjunct therapeutic effects on infectious pneumonia by acting on the core target FCER1A.

#### 3.3.2. Molecular Docking and Molecular Dynamics Simulation

To further investigate the binding capacity of the selected compounds to the target, molecular docking and molecular dynamics simulations were conducted for FCER1A in complex with pyrogallol and tectorigenin. Molecular docking results (Figure 10B) revealed that both pyrogallol and tectorigenin interact with the extracellular immunoglobulin-like domain of FCER1A. Specifically, the residues involved in the docking poses (e.g., CYS51 and GLU58 for pyrogallol; GLN182, HIS199, and ARG202 for tectorigenin) are all located within this domain, which is critical for FCER1A to recognize and bind its ligand, immunoglobulin E (IgE). Tectorigenin exhibited a more favorable binding energy (−7.0 kcal/mol), suggesting a more stable interaction and higher binding affinity for FCER1A. Functional prediction analysis indicates that these compounds, particularly tectorigenin, may directly hinder IgE binding to FCER1A by competitively occupying the IgE binding site or through steric hindrance. Consequently, this interaction is anticipated to suppress the mast cell/basophil activation signaling cascade triggered by the IgE-FCER1A complex, suggesting a promising therapeutic potential. Additional docking data are provided in Supplementary Table S1. Molecular dynamics simulations showed that the FCER1A–pyrogallol complex reached equilibrium after 20 ns with RMSD fluctuating around 2.9 Å, while the FCER1A–tectorigenin complex stabilized after 90 ns, exhibiting RMSD variations around 3.9 Å (Figure 10C), suggesting both systems formed stable complexes. Analysis of the radius of gyration (Rg) and solvent accessible surface area (SASA) showed only minor fluctuations during the simulation (Figure 10D,E), indicating conformational adjustments and moderate changes in the binding micro-environment upon ligand binding. Hydrogen bond analysis indicated that the FCER1A–pyrogallol complex formed 0–8 hydrogen bonds with an average of 3, while the FCER1A–tectorigenin complex maintained 0–5 hydrogen bonds with an average of 4 (Figure 10F), demonstrating favorable hydrogen bonding interactions with the target protein. The overall low root mean square fluctuation (RMSF) values (mostly below 4 Å) suggested limited residual fluctuations and high structural stability of the complexes (Figure 10G). In summary, both pyrogallol and tectorigenin form stable complexes with FCER1A characterized by substantial hydrogen bonding, indicating strong binding capabilities to this target protein.

## 4. Discussion

Infectious pneumonia represents a leading cause of mortality globally from respiratory tract infections, for which early diagnosis and effective treatment remain persistent challenges and focal points in both clinical and basic research [25]. This study integrated multi-omics data, machine learning algorithms, animal model experiments, and computational pharmacology to systematically investigate the immunopathological mechanisms of infectious pneumonia, identifying *FCER1A* as a pivotal gene with potential diagnostic and therapeutic value. Furthermore, two natural compounds capable of targeting *FCER1A* were predicted and preliminarily validated, offering novel insights for adjunct therapeutic strategies against infectious pneumonia.

It is well-established that the *FCER1A* gene encodes the high-affinity immunoglobulin E receptor alpha chain (FcεRIα), which serves as a core component of the FcεRI receptor [26,27]. This receptor belongs to the immunoglobulin superfamily and predominantly exists in two structural forms: the tetramer (αβγ_2_) expressed on mast cells and basophils, comprising α, β, and two γ subunits, and the trimer (αγ_2_) lacking the β subunit, primarily found on antigen-presenting cells and related cell types [28,29,30]. Specifically, the α subunit mediates the specific recognition of the Fc region of IgE, the β subunit functions in signal amplification, and the γ subunit initiates downstream signal transduction via its ITAM motif [31]. Notably, substantial differences exist between the human and mouse FcεRI systems. Humans co-express both tetrameric and trimeric forms, with the β subunit being non-essential in the human system, whereas mice predominantly express the tetrameric form, and the β subunit is critical for receptor expression and signal transduction in this species [28,31]. This divergence extends to the expression profiles: human FcεRI is broadly expressed across diverse immune cells, while murine expression is confined to mast cells and basophils [26,28]. Moreover, FcεRI demonstrates marked functional specificity across distinct human cell types. For instance, mast cells and basophils employ the tetrameric receptor to mediate acute allergic responses, whereas antigen-presenting cells utilize the trimeric form to initiate adaptive immune responses and contribute to the chronic persistence of allergic reactions [28,32,33,34,35]. Furthermore, FcεRI on the surface of cells such as platelets and eosinophils contributes to the modulation of inflammatory processes and tissue injury through distinct mechanisms. These differences exert significant influences on immune response mechanisms and therapeutic strategies [32]. The functional divergence between tetramers and trimers underlies the concomitant acute and chronic characteristics of human allergic responses, which also elucidates why murine models inadequately recapitulate the full complexity of human allergic diseases. Finally, genome-wide association studies (GWAS) have identified *FCER1A* as a key genetic susceptibility locus for serum IgE levels. Its single nucleotide polymorphisms (e.g., rs2251746 and rs2427837) can impair transcription factor binding (e.g., GATA-1), thereby reducing FCER1A promoter activity and FcεRIα receptor expression, which consequently modulates IgE levels [31,32] and significantly elevates the risk of developing allergic disorders such as asthma and atopic dermatitis [36]. Moreover, FCER1A exhibits dysregulated expression in autoimmune diseases like systemic lupus erythematosus and specific cancers (e.g., breast and lung cancers), suggesting its broader role in immune regulation [37,38]. Notably, the newly identified functions of the FcεRI trimer in tumor immunity have unveiled potential avenues for future research [28]. The expression of this gene is under the fine-tuned regulation of signaling pathways such as IL-4/STAT6, and targeted interventions against its downstream signaling have been successfully applied in treating severe asthma and chronic spontaneous urticaria, affirming the pivotal value of FCER1A as a critical immune marker and therapeutic target [26,39].

**Figure 11:**
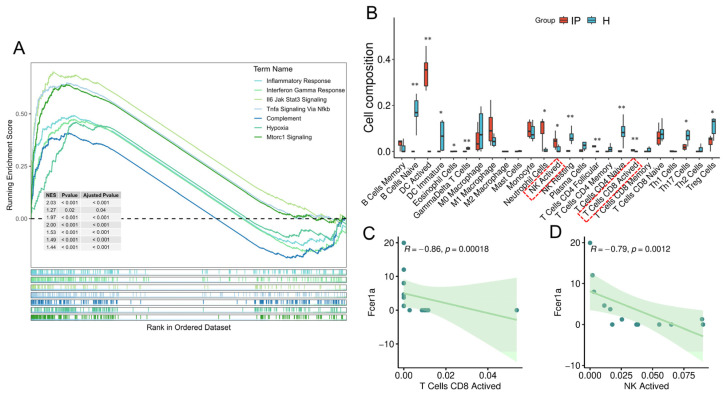
Putative mechanism of *FCER1A* inhibition in the pneumonia group. (**A**) GSEA of transcriptome in mouse lung tissues. (**B**) Boxplots illustrating the distribution of immune cell composition across the control group and the pneumonia group, as predicted by CIBERSORT analysis. (**C**) Correlation scatter plot of *Fcer1a* expression versus the infiltration level of activated CD8^+^ T cells. (**D**) Correlation scatter plot of *Fcer1a* expression versus the infiltration level of activated NK cells. * *p* < 0.05, ** *p* < 0.01 vs. healthy control group.

However, in the context of infectious pneumonia—a prototypical hyperinflammatory disorder under investigation in this study—we consistently observed a significant downregulation of *FCER1A*, a gene intricately associated with allergy and immune regulation. This finding aligns with a prior bioinformatics study that also identified *FCER1A* as a key gene and reported its significant downregulation in the disease group of respiratory syncytial virus-induced pneumonia [40]. This expression pattern diverges from the established understanding derived from allergic diseases, suggesting the potential existence of a unique and not yet fully elucidated regulatory mechanism in infectious pneumonia. Functional enrichment analysis of differentially expressed genes from the GSE103119 dataset revealed that the robust inflammatory and interferon response triggered by infectious pneumonia, particularly the marked activation of the IFN-γ signaling pathway, may be a critical factor suppressing *FCER1A* transcription. This discovery was further corroborated by GSEA enrichment analysis of the mouse lung tissue transcriptome (Figure 11A). IFN-γ is known to be primarily produced by activated NK cells and cytotoxic T lymphocytes (e.g., CD8^+^ T cells) [41]. Immune infiltration analysis in this study demonstrated a significant increase in the infiltration levels of these two cell populations in the lung tissue of the mouse pneumonia model (Figure 11B), both of which exhibited a significant negative correlation with *FCER1A* expression (*p* < 0.01; Figure 11C,D). Although the direct regulatory relationship between *FCER1A* and the IFN-γ signaling pathway has not been fully elucidated, we propose a speculative mechanism based on the aforementioned results: Pneumonia infection triggers a potent inflammatory response (e.g., elevated levels of IL-6, IL-1β, and TNF-α), initiating a predominantly type 1 immune response that subsequently recruits and activates NK cells and cytotoxic T cells. These cells secrete substantial amounts of IFN-γ, which may directly or indirectly suppress *FCER1A* transcription, potentially through the activation of downstream transcription factors such as STAT1, ultimately leading to reduced protein expression. This hypothesis also offers an explanation for the transient alleviation of allergic symptoms (e.g., asthma) observed in some patients following viral infections, suggesting that a robust type 1 response may temporarily suppress the type 2 pathway [42]. Furthermore, this hypothesis provides a novel perspective for understanding the aberrant expression of immunoregulatory genes during infection, though its validity necessitates further experimental verification.

In summary, within the context of infectious pneumonia, the downregulation of *FCER1A* expression does not represent a paradoxical phenomenon but rather constitutes a crucial negative immunoregulatory mechanism. This mechanism aims to suppress IgE-mediated overactivation of mast cells and basophils, thereby preventing inflammatory mediators such as histamine released via this pathway from exacerbating pulmonary inflammation and tissue damage. Such an adaptive response facilitates the reallocation of immune resources toward specific anti-infective effector mechanisms predominantly driven by type 1 responses, ultimately achieving pathogen clearance while maintaining immune homeostasis and preventing lethal immunopathological damage [43]. Building upon this foundation, we further employed a reverse network pharmacology approach to screen two natural compounds, pyrogallol and tectorigenin, as potential ligands targeting this molecule. Molecular docking analysis revealed favorable binding affinity of both compounds to FCER1A, with tectorigenin exhibiting a lower binding energy (−6.7 kcal/mol), suggesting potentially superior binding stability. Although direct evidence for the application of these compounds in treating infectious pneumonia is currently lacking, their documented anti-inflammatory and antimicrobial properties align with the complex immunopathology of infectious pneumonia, indicating their potential as adjunct therapeutic agents.

The principal innovations of this study are manifested in several key aspects. First, we implemented an integrated multi-database and machine learning cross-validation strategy to systematically identify molecular biomarkers associated with infectious pneumonia. By integrating transcriptomic data from multiple independent databases including GEO and employing three distinct machine learning algorithms—LASSO regression, random forest, and SVM-RFE—for feature gene selection, we significantly enhanced the robustness and reproducibility of biomarker identification, effectively overcoming the limitations of conventional single-method approaches that often yield unstable results with poor generalizability. Second, utilizing a *P. aeruginosa*-induced murine pneumonia model, we further validated the altered expression of the pivotal gene *FCER1A* at the in vivo level and demonstrated its correlations with pathological phenotypes and immune parameters, thereby reinforcing the credibility of our findings and their potential clinical translational value. Finally, by combining reverse network pharmacology with molecular simulation techniques, we identified potential natural compounds targeting FCER1A from a computational biology perspective and characterized their binding properties through molecular docking and molecular dynamics simulations, thereby providing novel insights into adjunctive therapeutic strategies for infectious pneumonia and establishing a feasible avenue for drug repurposing and rediscovery.

Nevertheless, this study has several notable limitations that warrant consideration. First, although the analysis of public transcriptomic data yielded valuable insights, the relatively modest sample size and incomplete clinical contextual information collectively compromise the generalizability and robustness of the findings. More importantly, due to substantial interspecies differences between human and murine FcεRI systems in receptor architecture, cellular distribution, and immune functions, experimental data derived from mouse models may not accurately recapitulate the human-specific functional divergence of tetrameric and trimeric forms or their complex regulatory mechanisms in adaptive immune modulation, thereby limiting the translational potential of our conclusions. Second, while transcriptomic profiling of bulk samples (e.g., whole blood and lung tissue) provides an overview of global immune status, the inherent cellular heterogeneity of these tissues precludes discrimination between gene expression differences arising from shifts in specific cell population proportions versus genuine alterations in intrinsic expression levels—a limitation that may obscure or dilute signals from critical cellular subsets. Future investigations should employ multi-center prospective cohort designs integrated with high-resolution technologies such as single-cell RNA sequencing to rigorously validate and dissect the immunologic mechanisms preliminarily identified herein at the level of distinct cell types. Finally, although the compounds identified through reverse pharmacology (e.g., pyrogallol and tectorigenin) demonstrate promising binding affinity at the molecular level, their in vivo efficacy, acute/chronic toxicity, metabolic stability, tissue distribution, and other pharmacokinetic/toxicological properties remain experimentally unvalidated, representing a significant gap toward clinical application that must be addressed in subsequent research.

## 5. Conclusions

In summary, by integrating bioinformatics and machine learning approaches, this study revealed that *FCER1A* expression is significantly downregulated in infectious pneumonia and demonstrates robust diagnostic performance. Animal experiments further validated that *FCER1A* expression levels are closely associated with pulmonary tissue damage and inflammatory responses, potentially through mechanisms involving activation of the IFN-γ signaling pathway. Furthermore, the natural compound tectorigenin was identified as a high-affinity binder of *FCER1A*, showing promising potential for development as a targeted therapeutic agent. Collectively, these findings identify a novel therapeutic target and provide a candidate lead compound for the diagnosis and treatment of infectious pneumonia.

## Data Availability

The original contributions presented in the study are included in the article/Supplementary Materials, further inquiries can be directed to the corresponding authors.

## Supplementary Materials

The following supporting information can be downloaded at: https://www.mdpi.com/article/10.3390/genes16111294/s1, Table S1: Parameters of proteins for visual screening.

## Abbreviations

The following abbreviations are used in this manuscript:

*FCER1A*	Fc epsilon receptor Ia
CAP	Community-acquired pneumonia
SCAP	Severe community-acquired pneumonia
RSV	Respiratory syncytial virus
*P. aeruginosa*	Pseudomonas aeruginosa
DEGs	Differentially expressed genes
PPI	Protein–protein interaction
LASSO	Least Absolute Shrinkage and Selection Operator
SVM-RFE	Support Vector Machine-Recursive Feature Elimination
ROC	Receiver operating characteristic
AUC	Area under the curve
FDR	False discovery rate
GSEA	Gene Set Enrichment Analysis
Tregs	Regulatory T cells
RMSD	Root mean square deviation
Rg	Radius of gyration
SASA	Solvent accessible surface area
RMSF	Root mean square fluctuation
TCM	Traditional Chinese Medicine
ANOVA	Analysis of variance
SD	Standard deviation
CFU	Colony-forming unit
PBS	Phosphate-buffered saline
HRP	Horseradish peroxidase
TSA	Tyramide signal amplification
DAPI	4′,6-Diamidino-2-phenylindole
RNA-seq	RNA sequencing
PCA	Principal component analysis
