# Supplementary material for: FCER1A Downregulation in Infectious Pneumonia: A Multi-Modal Study Combining Bioinformatics, Animal Models, and Reverse Pharmacology
Source: Genes (Basel). 2025 Oct 31;16(11):1294. doi: 10.3390/genes16111294 (PMC12652099; doi:10.3390/genes16111294)
Supplement: Supplementary file 1 [file genes-16-01294-s001.zip › genes-3962231-supplementary.pdf]

Supplementary Materials

FCER1A Downregulation in Infectious Pneumonia: A Multi-Modal Study Combining Bioinformatics, Animal Models, and Reverse Pharmacology

Yuan Cai [1], Xiaolong Feng [1], Mengxiong Xiao [2,*], Qian Li [3], Xinru Tao [1], Penghui Li [1]

[1] Institute of Innovative Chinese Medicine, Hunan Academy of Chinese Medicine, Changsha 410013, China; tcmyuanyuan@163.com (Y.C.)
[2] Experimental Research Center, China Academy of Chinese Medical Sciences, Beijing 100700, China
[3] College of Traditional Chinese Medicine and Health, Nanfang College Guangzhou, Guangzhou 510970, China
* Correspondence: xiaomengxiong25@163.com

Materials and methods

1.Cohort Characteristics of the GSE103119 and GSE228541 Datasets

The GSE103119 dataset comprised 152 hospitalized patients with community-acquired pneumonia (CAP) and 20 age-, sex-, and ethnicity-matched healthy controls. Demographic data revealed an overall median age of 5.1 years (IQR 2.1–8.0) in the CAP patient group, compared to 3.6 years (IQR 1.1–10.9) in the healthy control group. Comparative analysis showed that patients with Mycoplasma pneumoniae infection had the highest median age of 8.0 years (IQR 4.6–11.5), which was significantly greater than the median age of 3.6 years (IQR 1.5–6.3) in the viral infection group ($p < 0.001$). The male-to-female ratio was 75:77 in the pediatric CAP cohort and 13:7 in the healthy controls, with no statistically significant differences in sex or ethnicity distribution observed across the pathogen-based subgroups. Etiological testing identified sole respiratory viral infection as the most prevalent category (51%, 78/152), with rhinovirus/enterovirus (46%), respiratory syncytial virus (15%), parainfluenza virus (12%), and human metapneumovirus (11%) being the predominant viruses. Mycoplasma pneumoniae infection accounted for 28% (42/152) of cases, while pyogenic bacterial infection constituted 11% (16/152), primarily driven by Streptococcus pneumoniae (10 cases). Additionally, no causative pathogen was detected in 11% (17/152) of the patients. Among the 147 patients tested for viruses, the viral positivity rate was 67% (98 cases), with 22% of these representing co-infections; adenovirus and bocavirus were exclusively detected in mixed infections, and Staphylococcus aureus was identified as a sole pathogen in only 2 cases. The GSE228541 dataset included 14 CAP patients and 15 healthy controls for analysis. Patients were significantly older than controls (median age 78.0 vs. 55.0 years, $p<0.001$), whereas no significant differences were found in sex or body mass index (BMI) between the groups. Regarding comorbidities, malignancy was more frequent in the patient group (35.7%), whereas hyperlipidemia was more common in the control group (37.5%); neither chronic kidney disease nor immunocompromised status was present in either cohort. Clinical outcomes indicated that all patients survived, with a median hospital stay of 13.5 days.

2.Machine learning

For the machine learning analysis, the input was a normalized gene expression matrix obtained from the preprocessed GSE103119 dataset. In this matrix, rows represented samples and columns corresponded to the initial features (i.e., the 95 candidate genes derived from the protein-protein interaction network). The sample labels were

assigned based on the corresponding disease status (infectious pneumonia group vs. healthy control group). The specific training and feature selection procedures for each model were as follows: 1. LASSO Regression: The optimal regularization strength parameter (lambda.min) was automatically determined via 10-fold cross-validation. Using this parameter, LASSO performed feature selection by applying L1 regularization to shrink the coefficients of the majority of irrelevant or redundant genes to zero. Ultimately, all genes with non-zero coefficients were retained as key genes identified by this model. 2. SVM-RFE (Support Vector Machine-Recursive Feature Elimination): This algorithm commenced with the full feature set containing all 95 genes. In each iteration, a Support Vector Machine (SVM) model was trained using the current feature set. The importance of each feature was then ranked based on the model's weights (e.g., the square of the coefficients). The feature(s) with the lowest importance ranking were subsequently removed. This process was iteratively evaluated using 5-fold cross-validation, with the average classification accuracy serving as the evaluation metric, to ultimately identify the minimal optimal gene subset that achieved the highest performance. 3. Random Forest: We constructed a random forest model comprising 500 decision trees. The importance of each gene was quantified by calculating its mean decrease in accuracy score based on the out-of-bag (OOB) data. A higher score indicates a greater contribution of the gene to correct classification. Subsequently, all genes were ranked in descending order based on this importance score, and the top-ranked genes were selected as the key features output by this model. By integrating the selection results from the three algorithms described above, we derived a final list of key genes, thereby enhancing the robustness and reliability of the biomarker discovery.

3.Data filtering and analysis of transcriptomics analysis

Total RNA was isolated using TRIzol reagent (Thermo Fisher Scientific, 15596018). RNA integrity was assessed with a Qubit 3.0 Fluorometer (Thermo Fisher Scientific, Q33216) and Agilent 5300 Fragment Analyzer (Agilent Technologies, M5311AA), and only samples with RNA integrity number (RIN) > 7.0 were selected for downstream analysis. For library preparation, mRNA was purified from 2 μg total RNA through two rounds of selection using mRNA Capture Beads 2.0 (Yeasen Biotech, 12629ES). Purified mRNA was fragmented in magnesium-based buffer (Yeasen Biotech, 12340ES97) at 94°C, followed by first-strand cDNA synthesis with reverse transcriptase and second-strand synthesis using E. coli DNA polymerase I, RNase H, and dUTP solution. The fragmented cDNA underwent end repair, A-tailing, and ligation of Illumina dual-index adapters. PCR amplification was performed for 14

cycles (98°C for 1 min initial denaturation; 98°C for 10 sec, 60°C for 30 sec, 72°C for 30 sec per cycle; final extension at 72°C for 5 min) using high-fidelity DNA polymerase. Libraries with insert sizes of 400 ± 50 bp were purified with Hieff NGS DNA Selection Beads (Yeasen Biotech, 12601ES75) and sequenced on an Illumina NovaSeq X Plus platform (LC-Bio Technology) with 2 × 150 bp paired-end (PE150) configuration. Raw sequencing reads were filtered using Cutadapt (v1.11) to remove adapter-containing sequences, polyA/G tracts, reads with >5% ambiguous bases (N), and low-quality reads (Q-score ≤ 20). Data quality was verified with FastQC (v0.11.9) to assess Q20/Q30 scores and GC content. Clean data were deposited in the NCBI GEO/SRA databases under accession numbers GEO: < GEO accession > and SRA: < SRA accession >. Clean reads were aligned to the reference genome of the target species using HISAT2 (v2.2.1) with parameters allowing ≤ 2 mismatches and ≤ 20 multi-mapping positions per read. Splice junction databases were incorporated to enhance alignment accuracy. Transcript quantification was performed with StringTie (v2.1.6) and Ballgown to calculate FPKM (fragments per kilobase of transcript per million mapped reads) values. A comprehensive transcriptome was reconstructed by merging all sample-specific assemblies.

4.Molecular dynamics simulation

In this study, molecular dynamics (MD) simulations were performed using GROMACS 2022. Force field parameters were obtained using the GROMACS pdb2gmx tool and the AutoFF web server. The AMBER14SB force field was applied to the receptor protein, while the GAFF2 force field was used for the ligand. The system was solvated in a cubic box of TIP3P water molecules with a side length of 1 nm. Ions were then added using the gmx genion tool to neutralize the system. Long-range electrostatic interactions were treated using the Particle Mesh Ewald (PME) method with a cutoff radius of 1 nm. All bond constraints were applied using the SHAKE algorithm. An integration time step of 1 fs was employed, and the dynamics were propagated using the Verlet leapfrog algorithm. Prior to production MD simulations, the system was subjected to energy minimization. This process involved 3000 steps of steepest descent followed by 2000 steps of conjugate gradient minimization, carried out in three stages: (1) with the solute fixed and water molecules relaxed; (2) with counterions fixed; and (3) with no positional restraints applied to the entire system. The production simulation was conducted under an NPT ensemble at 310 K for a total duration of 100 ns. Trajectory analysis was performed using GROMACS tools including gmx rmsd, gmx rmsf, gmx hbond, gmx gyrate, and gmx sasa to evaluate the root mean square deviation (RMSD), root mean square fluctuation (RMSF), number of hydrogen bonds, radius of gyration (Rg), and solvent accessible surface area (SASA), respectively.

Supplementary Tables

Table S1. Parameters of proteins for visual screening.

Compound	Protein	PDB ID	Binding energy (kcal/mol)	Docking Box Center (x, y, z)	Docking Box Size (x, y, z)
Pyrogallol	FCER1A	8K7S	-5.0	(149, 129, 114)	(16, 16, 16)
Tectorigenin	FCER1A	8K7S	-6.7	(130, 116, 138)	(30, 27, 35)
